# The degree of inhomogeneity of the absorbed cell nucleus doses in the bronchial region of the human respiratory tract

**DOI:** 10.1007/s00411-019-00814-0

**Published:** 2019-10-05

**Authors:** Péter Füri, Árpád Farkas, Balázs G. Madas, Werner Hofmann, Renate Winkler-Heil, Gábor Kudela, Imre Balásházy

**Affiliations:** 1 Environmental Physics Department, MTA Centre for Energy Research, Konkoly-Thege Miklós út 29-33, Budapest, 1121 Hungary; 2 Department of Chemistry and Physics of Materials, University of Salzburg, Hellbrunnerstr. 34, 5020 Salzburg, Austria; 3 Eötvös Loránd University, Pázmány Péter sétány 1/C, Budapest, 1117 Hungary

**Keywords:** Lung cancer, Radon, Mucociliary clearance, Stochastic lung model

## Abstract

Inhalation of short-lived radon progeny is an important cause of lung cancer. To characterize the absorbed doses in the bronchial region of the airways due to inhaled radon progeny, mostly regional lung deposition models, like the Human Respiratory Tract Model (HRTM) of the International Commission on Radiological Protection, are used. However, in this model the site specificity of radiation burden in the airways due to deposition and fast airway clearance of radon progeny is not described. Therefore, in the present study, the Radact version of the stochastic lung model was used to quantify the cellular radiation dose distribution at airway generation level and to simulate the kinetics of the deposited radon progeny resulting from the moving mucus layer. All simulations were performed assuming an isotope ratio typical for an average dwelling, and breathing mode characteristic of a healthy adult sitting man. The study demonstrates that the cell nuclei receiving high doses are non-uniformly distributed within the bronchial airway generations. The results revealed that the maximum of the radiation burden is at the first few bronchial airway generations of the respiratory tract, where most of the lung carcinomas of former uranium miners were found. Based on the results of the present simulations, it can be stated that regional lung models may not be fully adequate to describe the radiation burden due to radon progeny. A more realistic and precise calculation of the absorbed doses from the decay of radon progeny to the lung requires deposition and clearance to be simulated by realistic models of airway generations.

## Introduction

In terms of detrimental health effects, the most important source of natural ionizing radiation is inhaled progenies of ^222^Rn (in the following called radon progeny). In fact, the inhalation of radon progeny is the second most important cause of lung cancer after smoking (World Health Organization (WHO) 2009). The proportion of lung cancers linked to radon is estimated to lie between 3 and 14% of total lung cancers, depending on the average radon concentration in a country and on the method of calculation (World Health Organization (WHO) 2009). Many epidemiological, especially case–control studies were carried out in the past to determine the correlation between radon levels in mines and the probability of lung cancer development (e.g. BEIR VI Report 1999; Hunter et al. 2013; Leuraud et al. 2011). These studies demonstrated that increased concentrations of inhaled radon progeny are associated with an increased risk of lung carcinomas. Additionally, to quantify the health risks for the general population, several studies have examined the relationship between radon levels lower than those in mines and the lung cancer risk in dwellings and at working places. Unfortunately, most of these studies hadn’t the statistical power to allow for the demonstration of an increased lung cancer risk (World Health Organization (WHO) 2009). Consequently, in an effort to use the information from individual studies, systematic reviews of the published papers were also performed (Lubin and Boice 1997; Lubin 1999; Pavia et al. 2003). These studies concluded that the radon-related risk of lung cancer varied appreciably from one study to another, which was mainly due to the different methodologies used in these individual studies to analyse the data. To avoid this heterogeneity, 3 pooled analyses, (1) Darby et al. (2005) including 13 individual European studies, (2) Krewski et al. (2005) including 7 individual North American studies and (3) Lubin et al. (2003) including 2 individual Chinese studies were performed. These investigations reported 8%, 11% and 13% increase in lung cancer risk per 100 Bq/m^3^ of radon gas concentration in air. The radon levels for the general population are much lower than those for miners. However, because the number of exposed individuals of the general population is much higher than that of miners, a considerable number of lung cancer cases can be caused by radon progeny in the general population. This fact demonstrates that the reduction of health risks associated with low levels of radon exposure is an important task of radiation protection.

There are two approaches to determine the radiation burden from the decay of inhaled radon progeny, the epidemiological and the dosimetric approaches. Unfortunately, there is a discrepancy between the results from these two ways of investigations. The dosimetric approach usually gives a higher dose and risk than the epidemiological studies. The factor of the difference was about 3 fifteen years ago (Stather 2004), but later the differences decreased. In the past, the epidemiological approach was preferred to quantify the relationship between radon exposure and lung cancer risk. In the ICRP 65 (1993) publication, the lifetime excess absolute risk for lung cancer due to exposure to radon and radon progeny was set to 2.8 × 10^−4^/WLM (working level month^1^). This value yielded rounded dose conversion coefficients of 5 mSv/WLM and 4 mSv/WLM for workers and the general public, respectively. Based on new accumulating data, the ICRP 115 (2010) publication revised the value of lifetime excess absolute risk for lung cancer due to exposure to radon and radon progeny to 5 × 10^−4^/WLM. The new value yielded rounded dose conversion coefficients of 12 mSv/WLM (workers) and 9 mSv/WLM (general public).

### Dosimetric approach

**Fig. 1:**
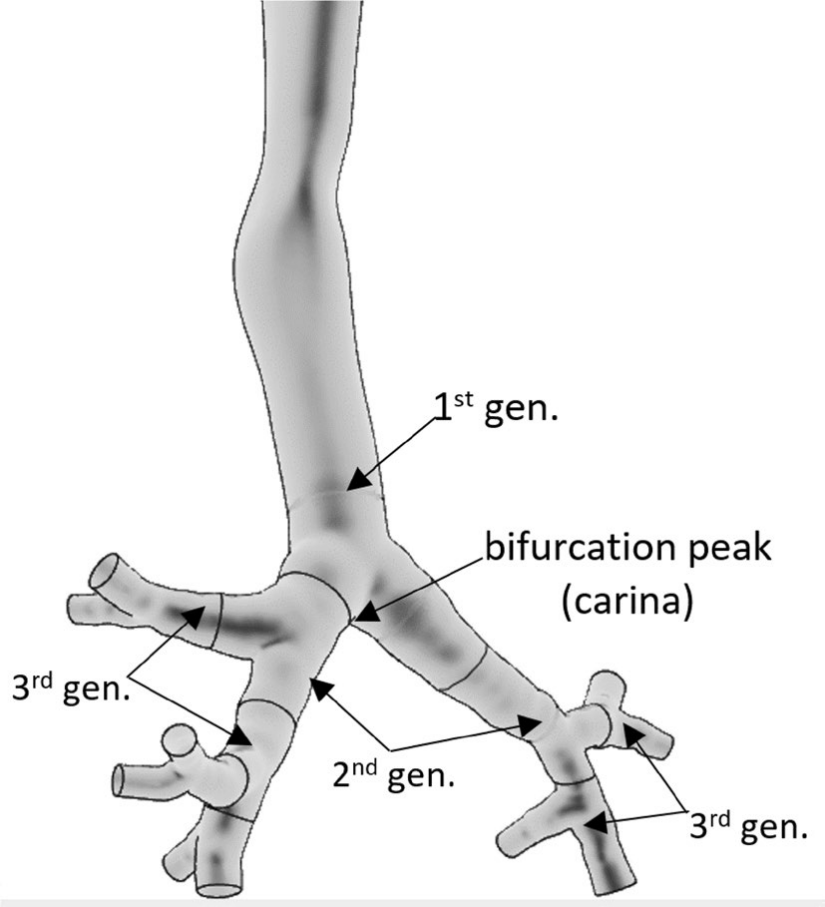
Segmentation of the airways into airway generations

For the doses to the lungs, only those progenies are relevant which deposit and decay in the airways after their inhalation. For the deposition and dose distribution calculations, the intrathoracic part of the human respiratory tract can be divided into airway generations (bifurcations). In the terminology used here, the trachea and the first half (which is closer to the trachea) of the left and right main bronchus constitute the first airway generation, while the second half of the main bronchi and the first half of their daughter branches constitute the second generation and so on (see Fig. 1).

The distribution of radon progeny deposited in the airways can be mathematically determined by special numerical models which may apply computational fluid dynamics (CFD) techniques (Farkas et al. 2011) or analytical methods. The advantage of the second type of approach is that it allows for a description of the deposition of radon progeny in the whole respiratory tract and not only in a certain segment of it (Hofmann 2011).

The two modelling approaches currently used to calculate lung doses from the decay of the short-lived radon decay products are: (1) semi-empirical compartment models (e.g., Radep/Imba based on the ICRP HRTM (human respiratory tract model) (ICRP 66 1994) and (2) deterministic or stochastic airway generation related models (Jacobi 1964; Haque and Collinson 1967; Harley and Pasternack 1972, 1982; Jacobi and Eisfeld 1980; Hofmann 1982; James 1988; Zock et al. 1996; Winkler-Heil et al. 2002, 2007; Hofmann et al. 2010).

The HRTM of the ICRP is one of the most often used biokinetic models (ICRP 66 1994). This model is a whole lung model but has a rather poor spatial resolution. As a result, deposition and clearance can be calculated only in one (mouth breathing) or two (nose breathing) extrathoracic compartments, two bronchial (BB—bronchial, bb—bronchiolar) compartments and one acinar compartment.

As a consequence of the poor spatial resolution, the HRTM is not able to describe the local airway generation-level deposition distribution of the inhaled radionuclides.

In addition, the HRTM includes a compartment-based clearance model with much longer clearance half-times than the half-life of the short-lived radon progeny. Therefore, this model does not allow for a realistic description of the airway generation-related radiation burden originating from the alpha decays of inhaled short-lived radon progeny. However, based on the results of previous modelling efforts (Balásházy and Hofmann 2000; Farkas et al. 2011; Baias et al. 2009) and the findings of early histopathological studies, it is known that the deposition distribution of inhaled particles can be highly inhomogeneous among different airway regions and even within a single airway bifurcation. For instance, Churg and Vedal (1996) observed high concentrations of deposited particles at the peaks (carina) of the bronchial airways (see Fig. 1).

### Lung models including a realistic airway structure

For an adequate description of the deposition and clearance of any inhaled particles in the human respiratory tract, lung models that include a realistic airway structure are needed. Because of the complex anatomy of the human lung, early models applied strongly simplified lung structures (e.g., Findeisen 1935; Weibel 1963). To better describe the human respiratory tract, a new way of lung modelling was developed in the 80s when Koblinger and Hofmann introduced the stochastic lung model (SLM) (Koblinger and Hofmann 1990; Hofmann and Koblinger 1990, 1992). This model was able to simulate the intra- and inter-subject variability of the human lung geometry using the Monte Carlo technique. The SLM model is very flexible in a sense that not only healthy but also diseased (asthmatic, emphysematic) lungs can be simulated (Füri et al. 2017). Since then, the model has been continuously improved. In the past decades, two deposition, clearance and radon dosimetry models have been developed on the basis of the original version of the SLM model that include a realistic airway structure. The IDEAL-DOSE code was created at the University of Salzburg, Austria (Hofmann et al. 2010), while the SLM-Radact code was developed at the Centre for Energy Research of the Hungarian Academy of Sciences, Hungary (Farkas et al. 2015; Füri et al. 2017). Although the basic structure of the two deposition models is similar, the Radact code was optimised to simulate the deposition of radon progeny and medical aerosols. For this purpose, a new region, the bronchiolus respiratorius (the first four acinar airway generations) was separated from the acinar airways. New sub-models were developed to simulate the deposition of hygroscopic and volatile particles and to calculate the surface of the simulated airways. While for dosimetric calculations, the IDEAL-DOSE code uses the method described by Mercer et al. (1991), the Radact code applies a completely new clearance and dosimetric model. These sub-models are unique tools, to investigate how the local (airway generation level) deposition and clearance affects the absorbed doses in the nuclei of the radiation-sensitive cells of the bronchial epithelium. The sub-models are described in the “Materials and methods” section.

The objective of the present study was to apply the Radact version of the SLM model, to investigate the inhomogeneity of absorbed doses to the cell nucleus due to deposition and clearance of inhaled short-lived radon progeny. For this purpose, the airway deposition and mucociliary clearance of the deposited radon progeny were quantified and absorbed dose distributions to the cell nucleus were calculated for an exposure of 1 WLM assuming a healthy sitting adult man in a typical indoor radiation environment.

## Materials and methods

### Breathing and aerosol parameters

For the simulation of radon progeny deposition, the breathing parameter values were taken from ICRP Publication 66 (ICRP 1994) (Table 1). Based on the ICRP recommendations, at the modelled level of exercise (sitting), only nose breathing was considered.

**Table 1:** Breathing parameter values used in the present work for an adult male while sitting

Physical activity	FRC (cm^3^)	VT (cm^3^)	f_B_ (min^−1^)
Sitting	3300	750	12

*FRC* functional residual capacity, *VT* tidal volume, *f*_*B*_ breathing rate

Based on data published by Marsh et al. (2005), spherical particles were considered with an activity median thermodynamic diameter (AMTD) of 0.8 nm (geometric standard deviation (GSD): 1.30) for unattached particles, and an activity median aerodynamic diameter (AMAD) of 230 nm with (GSD = 2.1) for attached particles. For the hygroscopic growth factor, values of 1 and 2 were assumed for unattached and attached particles, respectively. Parameters of radio-aerosols were adopted from a report of the United Nations Scientific Committee on the Effects of Atomic Radiation (UNSCEAR) (UNSCEAR 2000). For the ^218^Po, ^214^Pb and ^214^Bi activity concentration ratios, values typical for an average dwelling of 0.58, 0.44, and 0.29 were used, respectively, corresponding to an equilibrium factor of 0.4. The presented simulations were performed for an exposure of 1 WLM. Because the major fraction of short-lived radon progeny is already attached to particles of the surrounding air at the moment of their inhalation (Hopke 1989), in homes only 6% of the PAEC (potential alpha energy concentration) is due to unattached progenies (Haninger 1998). According to the International Commission on Radiation Units and Measurements (ICRU), 90% of the unattached PAEC in air is due to ^218^Po and the remaining 10% due to ^214^Pb (ICRU 2012). No unattached ^214^Bi and ^214^Po progenies are inhaled. However, in the respiratory tract, the deposited unattached ^218^Po may decay first to ^214^Pb, then to ^214^Bi and then to ^214^Po. Therefore, simulation of the disintegration of unattached ^214^Po is also necessary. There are both beta and alpha decays in the decay chain of ^222^Rn. However, because the biological effect of beta particles is much less significant than that of the two emitted alpha particles (a 6 MeV alpha particle from the decay of ^218^Po and a 7.69 MeV alpha particle from the decay of ^214^Po), the dose contributions of beta radiation was neglected in the present study.

### The Radact version of the stochastic lung model

To determine the deposition distribution to the cell nucleus and to simulate the clearance of the inhaled radon progeny, the Radact version of the SLM model was used (Farkas et al. 2015; Füri et al. 2017). In this version, the most important three deposition mechanisms (impaction, gravitational settling and diffusion) are simulated in stochastically generated, asymmetric airways. The geometric structure of the bronchial airways was reconstructed by Monte Carlo methods based on the database of Raabe et al. (1976). The acinar airways were built in a deterministic way based on the description given in Haefeli-Bleuer and Weibel (1988). For each simulation, hundred thousand individual pathways with different tube lengths and diameters, gravitational and branching angles were considered. In the present study, the empirical formula of Cheng (2003)  yielding the extrathoracic deposition was integrated into the model. The model was validated with the experimental data of Heyder et al. (1986) and Stahlhofen et al. (1989).

### Model for simulation of the fast mucociliary clearance

An important mechanism affecting the local distribution of the decays of radon progeny and the related biological effects (such as cell death and cell mutations) is the mucociliary clearance, also called fast clearance. This process involves transport of the deposited radon progeny towards the pharynx by the moving mucus layer which covers the bronchial airways. In the present simulations, the radon progeny was assumed to be transported immediately after their deposition with the velocity of the mucus layer and tracked until they decayed into ^210^Pb (which has a half-life of 22.3 years) or left the intrathoracic airways through the trachea. More specifically, the mucus layer was assumed to move with a velocity of 0.55 cm/min in the trachea (Cuddihy and Yeh 1988). The velocity of the mucus in all subsequent airway generations was assumed in the model to decrease by a factor of 0.67 generation by generation, from the trachea to the last bronchial airway (Hofmann and Sturm 2004). As a consequence, the mucus velocity (expressed in cm/min) in the *i*th generation was computed as 0.55 × (2/3)^*i*−1^. To demonstrate the effect of clearance, all decays occurring in the airway generation in which the radon progeny were originally deposited were associated with the deposition, while decays in an airway generation different from the one in which the radon progeny of interest was primarily deposited were assigned to clearance. Clearance time (the time interval needed for any radionuclide to be moved along the whole length of the investigated airway) for each airway generation depends on two factors: (1) the length of the airway, and (2) the mucus velocity in that airway. Based on the above mucus velocity values and the lengths of the airway branches, the present calculations yielded for the clearance times 19.1 min in the 1st airway generation, 9.8 min in the 2nd airway generation and 7.3 min in the 3rd airway generation. From this 3rd airway generation on, the clearance times increase significantly. However, in the first 8–10 airway generations, they are still shorter than the half-lives of the radon progeny. As a result, many of the deposited radon progeny will be transported by one or more airway generations upwards before decaying. The clearance times for the 8th–21st airway generations are much longer than the half-lives of the radon progeny ^218^Po (3.07 min), ^214^Pb (26.8 min) and ^214^Bi (19.9 min). As a consequence, the probability that these radioisotopes are cleared up with the mucus at least one airway generation—without reaching the ^210^Pb stage of the decay chain—is quite small. In other words, in the deeper part of the bronchial airways, the mucociliary clearance does not significantly influence the location of the radioactive decays which, as a result, remains similar to the location of deposition. This is consistent with the predictions of the IDEAL-DOSE particle deposition and clearance model of Hofmann and Winkler-Heil (2011).

### Model for calculation of absorbed doses to the cell nucleus

**Fig. 2:**
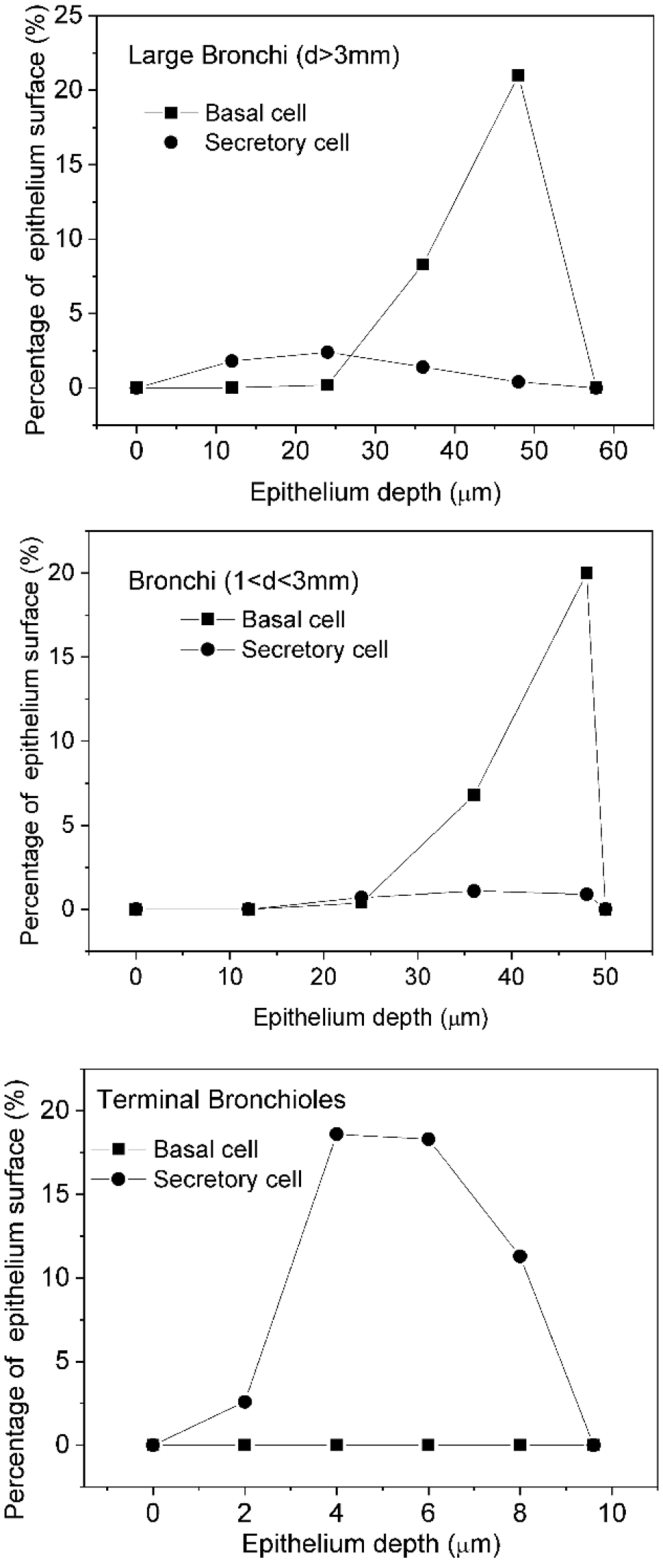
Depth distribution of the basal and secretory target cells in the epithelium of the large bronchi, bronchi and terminal bronchioles

In the dosimetric model of the Radact version of the SLM model, the airway structure was similar to the one used for deposition calculations. The initial positions of alpha particle-emitting isotopes corresponded to the deposition sites. The basal and secretory cells were placed according to Mercer et al. (1991) (Fig. 2).

The study of Mercer et al. (1991) specified the regions in the lung not by airway generation number but by the diameter of the airways. Therefore, it was considered in the present work that an airway is a large bronchus, if the diameter is greater than 3 mm. Similarly, a duct is a bronchus, if the diameter is less than 3 mm but greater than 1 mm, and a terminal bronchiole, if the diameter is less than 1 mm. Based on this classification, in the SLM model, the first 10 airway generations are considered as large bronchi, the 11th–13th airway generations as bronchi and the 14th–21st airway generations as terminal bronchioles.

**Fig. 3:**
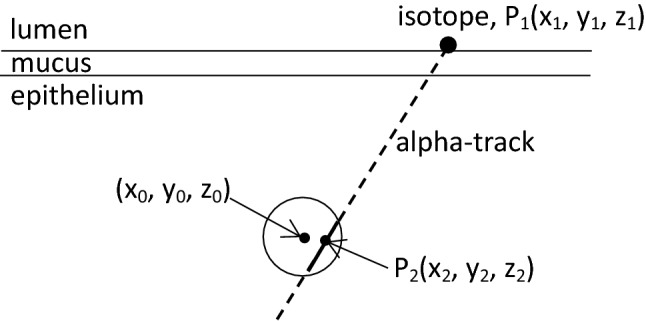
Schematic representation of alpha particle–cell nucleus interaction

Figure 3 demonstrates the methodology used for the simulation of the interaction of alpha particles with the nuclei of radiosensitive cells.

The alpha-emitting ^218^Po or ^214^Po isotopes were distributed randomly on the top of the airway-covering mucus layer. In Fig. 3, the coordinates of the ^218^Po or ^214^Po isotopes are *P*_1_(*x*_1_, *y*_1_, *z*_1_). To avoid boundary effects (loss of alpha particles which would exit the domain), the simulated cylinder was three times longer than the actual airway. The basal or secretory cells were placed bellow the middle of the analysed airway. In each airway generation, 25 different equidistant depths in the epithelium were simulated starting from the basal membrane to the top of the epithelium (epithelium–mucus interface). After both the basal and secretory cell nuclei and the alpha-emitting nuclides were positioned, a target point with coordinates *P*_2_(*x*_2_, *y*_2_, *z*_2_) was randomly selected inside the simulated cell nucleus. From these two points (*P*_1_ and *P*_2_), the following parametric equations of the alpha track can be created:

$$\begin{aligned} x = \left( {x_{2} - x_{1} } \right) \times t + x_{1} \hfill \\ y = \left( {y_{2} - y_{1} } \right) \times t + y_{1} \hfill \\ z = \left( {z_{2} - z_{1} } \right) \times t + z_{1} , \hfill \\ \end{aligned}$$

where *t* is a parameter. The nuclei of the basal or secretory cells were considered to be spheres. Thus, the surface of these cell nuclei can be described as:

$$\left( {x - x_{0} } \right)^{2} + \left( {y - y_{0} } \right)^{2} + \left( {z - z_{0} } \right)^{2} = r^{2} ,$$

where (*x*_0_, *y*_0_, *z*_0_) is the centre and *r* is the radius of the sphere (nucleus).

Solving the system of the above four equations yields the coordinates of the points, where the emitted alpha particles enter and exit the investigated cell nucleus. The probability that an alpha track includes *P*_2_ depends on the position of *P*_2_ within the sphere, depth of the nucleus in the epithelium and the position of the deposited radioisotope. Knowing the length of the simulated alpha particle pathway in the radiation-sensitive nucleus of the target cell, the absorbed energy can be determined using the alpha particle energy-range data calculated by the SRIM (specific range of ions in matter) code which is a commercially available code. Obviously, alpha particles may not enter the nucleus if their energy is entirely absorbed before reaching the nucleus, and they may enter but not exit the nucleus if their energy becomes zero within the nucleus. The above presented model provides the dose to the cell nucleus from one alpha particle track from a single randomly placed radioisotope.

To compute the average dose from one alpha particle, several radioisotopes must be considered and the corresponding absorbed cell nucleus doses averaged. Since the hit probability is a function of the radioisotope position (Crawford-Brown and Shyr 1987), a weighted average needs to be computed. For this purpose, in every airway, 50,000 ^218^Po and 50,000 ^214^Po isotopes were placed, and from each isotope one alpha particle track was simulated. The number of the simulated tracks (50,000 for each radioisotope and each of the 25 different depths in each bifurcation) resulted from an optimization process aiming at a reasonably low computer running time and sufficiently high statistical precision. After the determination of the average absorbed doses from a single alpha particle track at 25 different depths, the results were averaged. Since every depth has a different probability, this averaging was also weighted. Then, the weighted average dose for one alpha particle hit was multiplied with the total number of alpha decays originating from the progenies decayed in that airway generation, which depended on the deposition at the given macroscopic exposure (e.g., 1 WLM).

## Results and discussion

### Computed deposition fractions

**Fig. 4:**
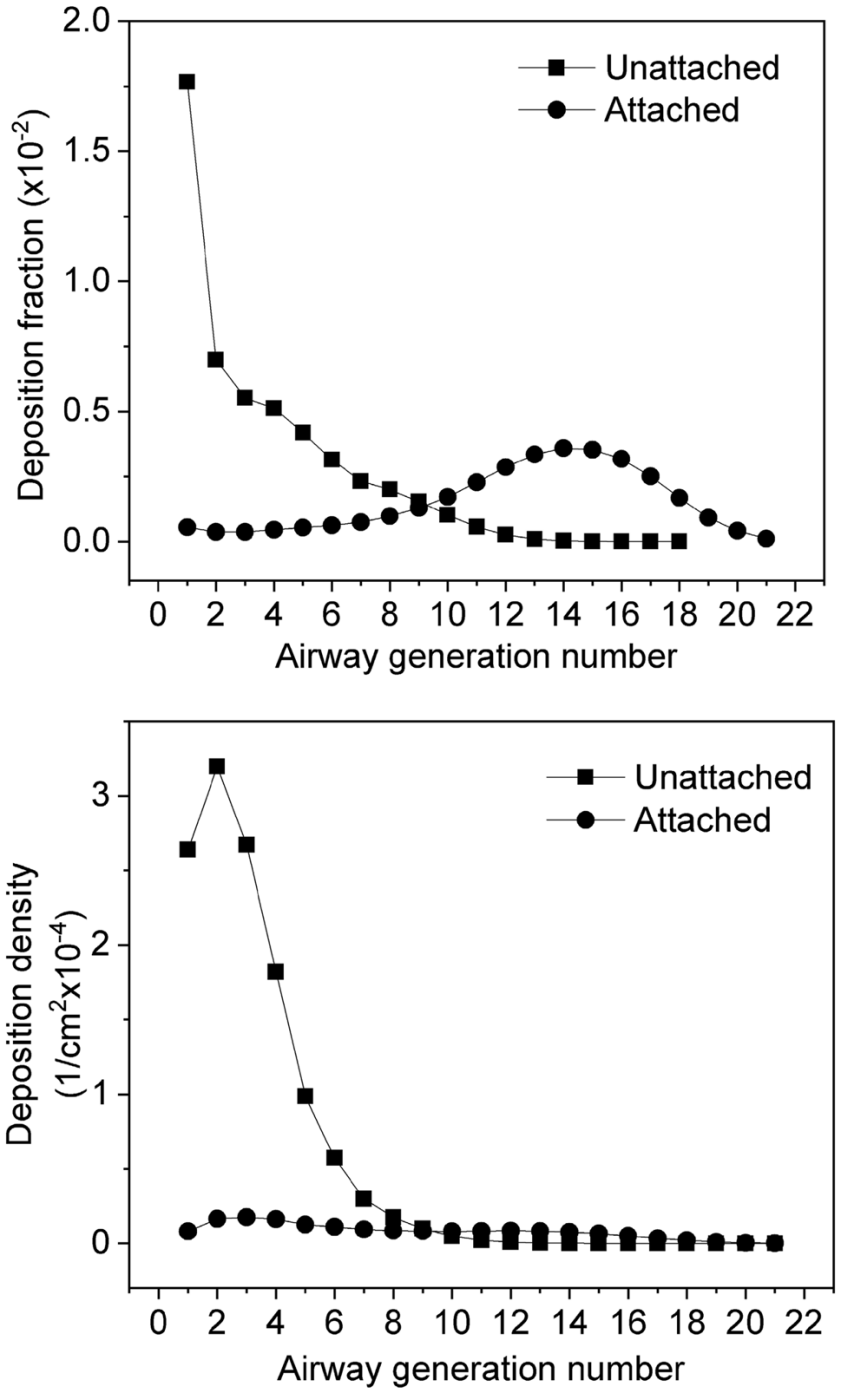
Deposition fractions and deposition densities of attached and unattached radon progenies in bronchial airways depending on airway generation number, for a sitting male with corresponding breathing rates, at an indoor radiation exposure of 1 WLM

In this study, deposition fractions were calculated as the ratio of the number of radioactive particles deposited in a given airway segment (e.g., airway generation) to the number of inhaled particles. Deposition density was defined as the ratio of the deposition fraction of a certain airway segment to the surface area of this airway segment. Figure 4 depicts the computed deposition fractions and deposition densities of unattached and attached radon progeny as a function of bronchial airway generation number assuming a breathing mode characteristic of sitting. As the figure reveals, the deposition fractions and their distributions for the investigated airway generations are quite different for unattached and attached radon progeny. The extrathoracic deposition fraction of the unattached progenies is very high (94.0%), and also high in the bronchial airways (airway generation number: 1–8, ICRP 66). For comparison, an extrathoracic deposition probability for the AMTD = 0.8 nm particles of 88.6% was calculated by (Winkler-Heil et al. 2007) using the IDEAL-DOSE code. The Radep/Imba based on the HRTM in this publication gives a somewhat lower extrathoracic deposition probability of 83.4% (Winkler-Heil et al. 2007). This is due to the fact that different empirical deposition formulas were used. Deposition fraction values of the unattached radon progeny within the large bronchial airways decrease monotonously with increase in generation number (see Fig. 4). The maximum of the deposition fraction was found in the largest bronchial airways. Although these airways show the largest diameters, the total surface of the branches belonging to these airways is the lowest. This means that even a small number of deposited progenies may result in high deposition density rates and activity densities, which is important for the occurrence of biological effects. Compared to the unattached progenies, the deposition fractions of the attached progenies are relatively low in the extrathoracic region (6.9%) and in the bronchial airways, because most of the inhaled attached progenies are exhaled without deposition (84%). The deposition fractions of the attached progenies increase until the 15th airway generation, then decrease towards the acinar airways (Fig. 4). Although a considerable number of attached progenies are deposited in the bronchiolar region (airway generations 9–21, ICRP 66), the deposition density values in this region are low (see the bottom panel of Fig. 4), because the total surface of these airways is large compared to the surface of the first eight airway generations. The difference between the deposition fractions of unattached and attached progenies demonstrates how important it is to know the size distributions of the radon progeny in the air of living and working places.

### Simulated dose rates to the cell nucleus

The doses absorbed by the cell nucleus depend on many parameters. The first and most important parameter is the number of alpha decays in the investigated airway generation. This number is usually decreasing with increasing number of airway generation. The second important parameter is the hit probability associated with a single decay. This probability increases significantly from the first to the last bronchial airway generation, because the diameters and lengths of the airways and the thickness of the mucus layer are all decreasing with increasing number of airway generation. The third parameter is the average energy absorbed in one target cell nucleus which in turn depends on the track length within the nucleus and the energy of the alpha particle.

Based on the ICRP Publication 66 (ICRP 1994), the most radiosensitive cells of the bronchial epithelium are the basal and secretory cells. Therefore, absorbed doses in the nuclei of these cells were considered in the present study. According to Mercer et al. (1991), secretory cells can be found in all regions of the bronchial airways, while basal cells can be found only in the large bronchi (airway diameter > 3 mm) and the bronchial region (airway diameter between 1 and 3 mm). In the terminal bronchioles, there are no basal cells and therefore, the absorbed dose in the basal cells is zero after the 14th airway generation. The depth distribution of the basal cells is quite different from the depth distribution of the secretory cells (Fig. 2). Basal cells can practically be found only in the deeper parts of the bronchial epithelium. Therefore, both the hit probabilities and the energy of the alpha particles reaching the radiation-sensitive cell nuclei are usually lower for basal than for secretory cells. Furthermore, the emitted alpha particles mostly lose all of their energy before they reach the basal cells. This explains why the absorbed doses are always much lower in the basal than in the secretory cells. It is worth mentioning that the doses for the two cell types differ because of their different depth distributions. At the same time, the ratio of absorbed doses originating from the decay of the locally deposited and up-cleared progeny is not affected by the different depths of these two cell types. The difference is caused only by the different number of the emitted alpha particles.

**Fig. 5:**
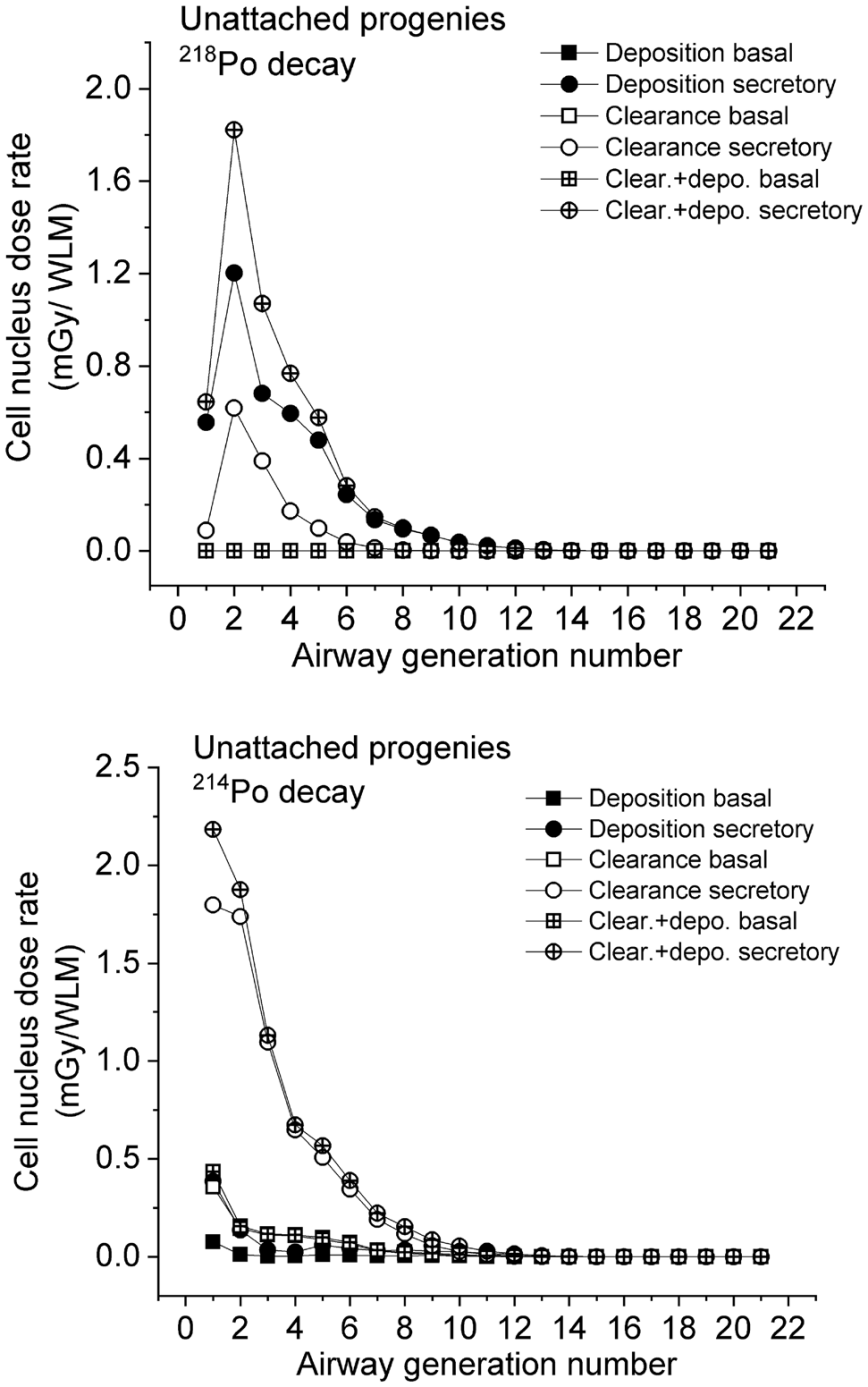
Absorbed doses to the cell nucleus in a healthy sitting adult male with corresponding breathing rates as a function of bronchial airway generation number, for the decays of unattached ^218^Po and ^214^Po progeny (^214^Po decays originate from the inhaled unattached ^218^Po and ^214^Pb)

**Fig. 6:**
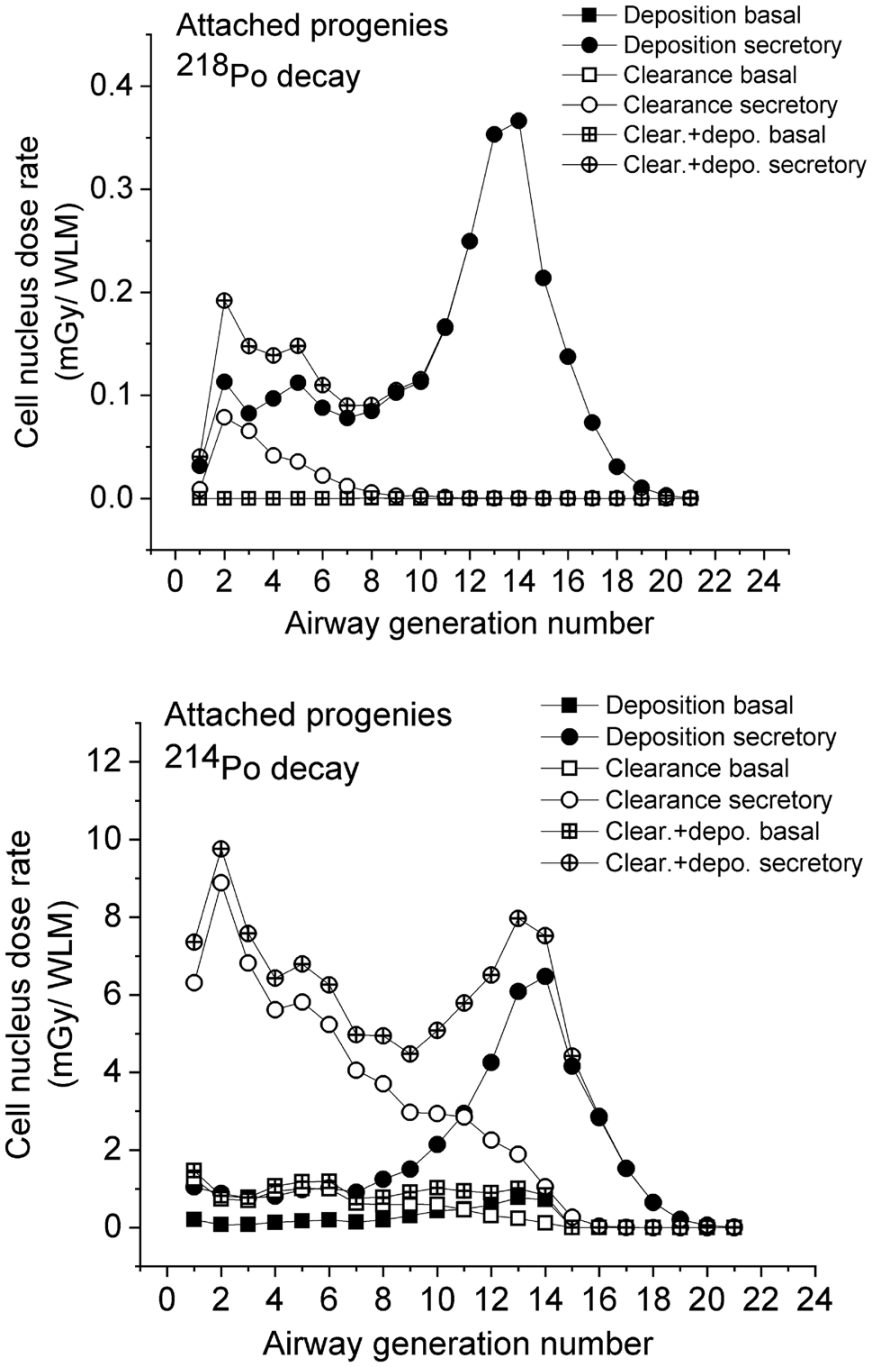
Absorbed dose rates to the cell nucleus in a healthy sitting male with corresponding breathing rates as a function of bronchial airway generation number, for the decays of attached ^218^Po and ^214^Po progeny

Figures 5 and 6 compare the dose rates absorbed by the cell nucleus as a function of the number of bronchial airway generation when the absorbed dose originates from radionuclides directly deposited to a certain airway, from radionuclides transported by mucociliary clearance from deeper airway regions, or from deposition and clearance (total), for decays of unattached and attached ^218^Po and ^214^Po, respectively. Both for unattached and attached progenies, the absorbed dose to the secretory cells in the bronchial airways (airway generation 1–8) from the decay of the ^218^Po progeny originates mostly from deposition, so the radioisotopes involved decay mostly in the same airway generation where they were originally deposited. This can be explained by the short half-life (3.05 min) of ^218^Po, which decays before being transported by the mucus to the neighbouring airway. As an opposite process, the absorbed dose due to the decay of the ^214^Po progeny originates mostly from clearance. This means that these decays occur mostly one or more airway generation upwards from the location of ^214^Po deposition. An important factor regarding the probability of radioactive decay is the available time. Except for the first three airway generations, it takes a longer time than the half-life of the radon progeny to move from an airway generation to another. As an effect of the long-lasting transport of the deposited progeny with the mucus layer, a considerable number of the inhaled ^218^Po, ^214^Pb or ^214^Bi radionuclides can decay to ^214^Po before reaching the trachea.

Figure 5 reveals that in the case of unattached progenies the maximum of total absorbed doses is in the 1st or 2nd airway generation. It is worth mentioning that in the first three airway generations, even if no unattached ^214^Po radionuclides are inhaled, the absorbed doses originating from the decays of the ^214^Po progeny (of the inhaled unattached ^218^Po and ^214^Pb) are higher than the doses originating from the decay of unattached ^218^Po. This is partly due to the higher initial alpha energy associated with the decay of ^214^Po (7.69 MeV) compared to the alpha energy associated with the decay of ^218^Po (6 MeV), and partly due to the fact that in the case of ^214^Po the inhaled unattached ^214^Pb also contributes to the absorbed dose.

Figure 6 shows the absorbed doses from the decays of attached ^218^Po and ^214^Po progeny. The dose distribution curves shown differ significantly from those the unattached progeny. This is a consequence of the different deposition distributions of the attached and unattached progenies. For the attached progenies, the maximum of total absorbed doses (deposition + clearance) due to the decay of the ^218^Po progeny is in the secretory cells of the 14th airway generation. This is the location where most of the attached progenies are deposited. In the bronchial airways (airway generation 1–8), the absorbed doses from the decays of the ^218^Po progenies are much lower than they are around the 14th airway generation. This can be explained by the thicker epithelium in the bronchial airways, that is, the deeper location of the sensitive cells. As a consequence, in the bronchial airways, most of the alpha emitters are distant from the target cells and the emitted 6 MeV alpha particles lose considerably energy before reaching the nuclei of the basal and secretory cells.

The situation is different for absorbed doses from the decay of the attached ^214^Po progeny. The interplay between the three different parameters influencing the doses to the cell nucleus (number of decays, hit probability and absorbed energy) results in maximum absorbed doses to the cell nucleus for the deposition and clearance around airway generation 2. The decays of the cleared-up particles (i.e., particles that were transported by mucociliary clearance from deeper airway generations) dominate in the first ten airway generations. In this region of the respiratory system, the clearance times are short enough for a significant number of the deposited ^214^Pb or ^214^Bi radioisotopes to be transported one or more airway generations upwards from the location of their deposition before decaying. In the case of the decays of the ^214^Po progeny and secretory cells, the emitted 7.69 MeV alpha particles have enough energy to reach the radiation-sensitive cells even in the beginning of the bronchial airways. The number of airways of the same generation is low in this region of the respiratory tract, so the number of decays in one airway will be high. As an outcome result, the absorbed doses to the cell nucleus can be high even for a small number of deposited radon progeny, low hit probability and low average absorbed energy.

The upper panels (on the decays of the ^218^Po progeny) of Figs. 5 and 6 reveal that the dose values in Fig. 5 are about five times higher than those in Fig. 6, although only 35% of the inhaled ^218^Po belongs to the unattached fraction. This is an effect of the different deposition distributions of attached and unattached progenies. The unattached radon progeny deposit with much higher probability in the bronchial airways than the attached radon progeny. This indicates that even a small number of inhaled unattached progenies can result in high absorbed doses in the large bronchial airways.

**Fig. 7:**
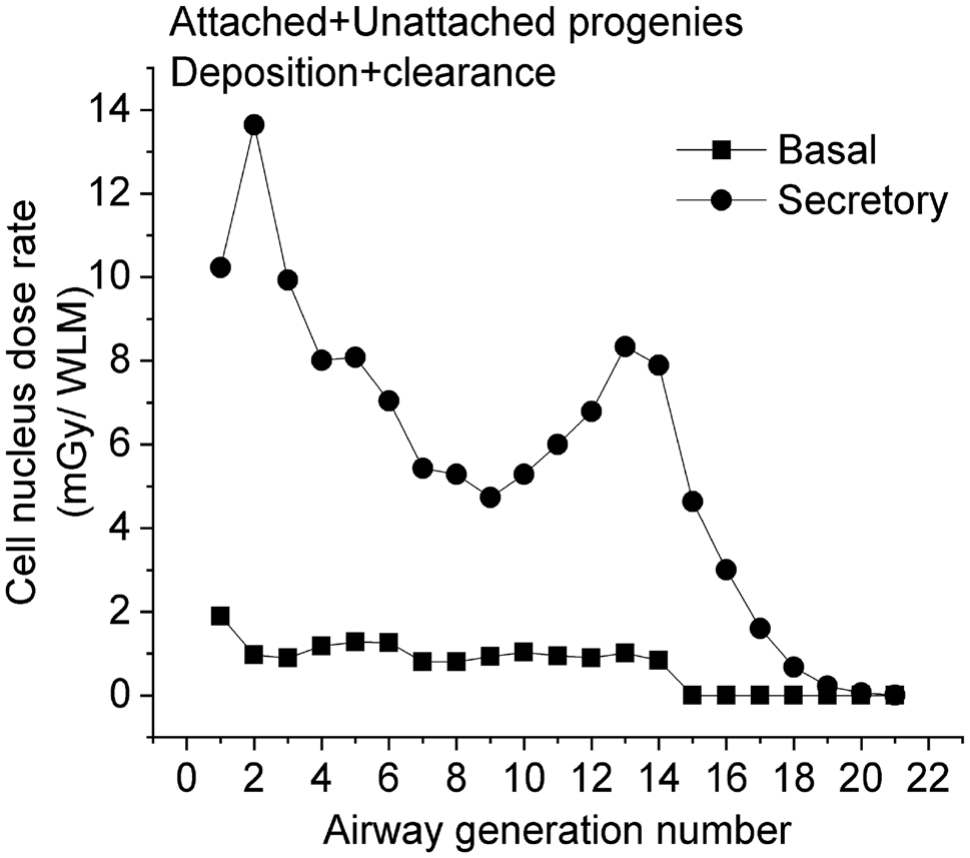
Total absorbed dose rate from the decays of the attached + unattached ^218^Po + ^214^Po radon progeny

Figure 7 shows the distribution of the total radiation dose (the decay of the attached + unattached ^218^Po + ^214^Po progeny), which includes—for secretory cells—a high and pronounced peak at the 2nd airway generation and a lower peak around the 13th airway generation. It is worth mentioning that the location of the first peak coincides with the site where most of the lung carcinomas were found (Auerbach et al. 1961; Kotin and Falk 1959; Macklin 1956; Veeze 1968; Auerbach and Garfinkel 1991; Saccomanno et al. 1996). The figure confirms the high degree of absorbed dose inhomogeneity within both the bronchial (BB, airway generation 1–8) and bronchiolar (bb, airway generation 9–15) airways. This inhomogeneity cannot be accounted for by the widely used HRTM model. The present results indicate that a regional level of dosimetry may not be sufficient when investigating the potential biological effects due to inhalation of radon and radon progeny.

### Comparison with dose rates predicted by other models

Based on the dosimetric approach including the ICRP HRTM lung model, the values of effective dose at an exposure of 1 WLM range from 10 to 20 mSv/WLM, depending on the exposure scenario (ICRP 66 1994; ICRP 115 2010). For example, using this model, Marsh et al. (2002) reported a dose conversion coefficient of 15 mSv/WLM for indoor radon exposure.

Hofmann and Winkler Heil (2011) calculated a dose conversion coefficient of 7.82 mSv/WLM with the IDEAL-DOSE code when averaging absorbed doses over all bronchial and bronchiolar airways, for typical indoor exposure conditions and a sitting adult male. The Radact version of the SLM model yielded 8.11 mSv/WLM when again absorbed doses for all bronchial and bronchiolar airways were averaged, at an equilibrium indoor equilibrium factor *F* = 0.4 and for a sitting adult male. This demonstrates that the results of the two models involving different dosimetric codes (Radact and IDEAL-DOSE) are similar but provide slightly smaller dose conversion coefficients than the HRTM model.

## Conclusions

The results of this study demonstrate that the Radact version of the SLM model can provide valuable information on the distribution of absorbed doses due to inhaled short-lived radon progeny at airway generation level. The results presented here reveal that deposition distributions of unattached and attached progenies are quite different in all characteristic regions of the respiratory tract. Radon progenies that are not attached to ambient particles are deposited mainly in the extrathoracic and the bronchial (airway generation 1–8, ICRP 66 1994) regions. By the same token, the extrathoracic deposition of the attached progenies is not dominant and the deposition fraction values are relatively low also in the bronchial airways, increasing with airway generation number until about the 15th airway generation. The absorbed doses are affected by several parameters (number of decays, hit probability, average absorbed energy) which are strongly variable both in the BB and bb regions of the HRTM. As a consequence, the absorbed doses to the cell nucleus are non-uniformly distributed within the bronchial region of the respiratory system both for the unattached and attached progenies. In the case of the unattached progeny, there is a pronounced peak of the absorbed dose rate at the beginning of the BB region, namely in the 1st–2nd airway generations. For the attached progeny, two peaks can be observed (the higher peak is at the 2nd and the lower peak is at the 13th–14th airway generations) and the majority of the absorbed doses originate from the decay of progeny deposited at deeper airway generations and transported upwards by mucociliary transport. Regarding the total absorbed doses (the decays of the unattached + attached, ^218^Po + ^214^Po progeny), the maximum is in the 2nd airway generation. The simulations performed by the Radact version of the SLM model show that simulation of radon progeny deposition and clearance in various airway generations can provide a possible explanation for the enhanced frequency of lung carcinomas found in the large bronchial airways.

The two main messages of this study are that: (1) absorbed doses in the lungs are strongly variable along the airway generations both within the BB and bb regions of the HRTM lung model and (2) clearance as a function of airway generation strongly affects absorbed dose rates. This suggests that for the investigation of the biological effects of radon and radon progeny inhalation (like the probability of lung cancer development), the analysis of absorbed doses just in the BB and bb regions of the lungs may not be sufficient.

## Footnotes

1 Working level (WL): any combination of the short-lived progeny in 1 L of air that results in the ultimate release of 1.3 × 10^5^ MeV of potential alpha particle energy. Working level month (WLM): cumulative exposure to an individual exposed at this concentration over a “working month” of 170 h, or at twice this concentration over half as long, etc. (World Health Organization (WHO) 2009).
